# Co-Administration of Fendiline Hydrochloride Enhances Chemotherapeutic Efficacy of Cisplatin in Neuroblastoma Treatment

**DOI:** 10.3390/molecules25225234

**Published:** 2020-11-10

**Authors:** Antonella Brizzolara, Patrizia Garbati, Serena Vella, Matilde Calderoni, Alessandro Quattrone, Gian Paolo Tonini, Mario Capasso, Luca Longo, Raffaella Barbieri, Tullio Florio, Aldo Pagano

**Affiliations:** 1 IRCCS AOU San Martino Polyclinic Hospital, 16132 Genova, Italy; antonella.brizzolara@yahoo.it (A.B.); tullio.florio@unige.it (T.F.); 2 Department of Experimental Medicine (DIMES), University of Genova, 16126 Genova, Italy; p.r.garbati@gmail.com (P.G.); calderoni.matilde@gmail.com (M.C.); raffaella.barbieri@edu.unige.it (R.B.); 3 Department of Laboratory Medicine and Advanced Biotechnologies, Institute of Hospitalization and Care of a Scientific Nature—Mediterranean Institute for Transplantation and Highly Specialized Therapies (IRCCS- ISMETT), 90127 Palermo, Italy; vellaserena@gmail.com; 4 Anemocyte S.r.l., 21040 Gerenzano, Italy; 5 Laboratory of Translational Genomics, Centre for Integrative Biology, University of Trento, 38123 Trento, Italy; alessandro.quattrone@unitn.it; 6 Neuroblastoma Laboratory, Pediatric Research Institute, The “Città della Speranza” Foundation, 35128 Padua, Italy; gp.tonini@irpcds.org; 7 Department of Molecular Medicine and Medical Biotechnology, University of Naples Federico II, 80145 Naples, Italy; mario.capasso@unina.it; 8 CEINGE Biotecnologie Avanzate, 80131 Naples, Italy; 9 SDN Research Institute Diagnostics and Nuclear, 80133 Naples, Italy; 10 Lung Cancer Unit, Division of Medical Oncology II, IRCCS San Martino Polyclinic Hospital, 16132 Genova, Italy; longo.luk@gmail.com; 11 Department of Internal Medicine (DIMI), University of Genova, 16126 Genova, Italy

**Keywords:** neuroblastoma, non-coding RNA, NDM29, fendiline hydrochloride, GD2

## Abstract

Despite significant improvement of neuroblastoma (NB) patients’ survival due to recent treatment advancements in recent years, NB is still associated with high mortality rate. In search of novel strategies to increase NB’s susceptibility to pharmacological treatments, we investigated the in vitro and in vivo effects of fendiline hydrochloride as an enhancer of cisplatin antitumor activity. To assess the modulation of fendiline treatment on cisplatin responses, we used in vitro (evaluating NB cell proliferation by XCELLigence technology and colony formation, and gene expression by RT-PCR) and in vivo (NB cell grafts in NOD-SCID mice) models of NB. NB cell treatment with fendiline induced the expression of the ncRNA NDM29, leading to cell differentiation and to the reduction of the expression of MDRs/ABC transporters linked to multidrug resistance. These events were correlated to higher NB cell susceptibility to cisplatin and, consequently, increased its cytotoxic potency. In vivo, this drug interaction causes an enhanced ability of cisplatin to induce apoptosis in NB masses, resulting in tumor growth reduction and prolonged animal survival rate. Thus, the administration of fendiline might be a possible novel therapeutic approach to increase cisplatin efficacy in aggressive and poorly responsive NB cases.

## 1. Introduction

Neuroblastoma (NB) is a pediatric cancer that arises from neural crest progenitor cells of the developing sympathetic nervous system. It usually onsets in the paraspinal sympathetic ganglia of the neck, chest, abdomen, or in pelvic ganglia, as well as within chromaffin cells of the adrenal gland medulla [1,2,3,4]. NB is the second most common solid tumor of childhood and typically affects children before school age, although in some cases it also may occur in young adolescents and adults [4,5,6]. In children with high-risk NB, the modest survival improvement has been recorded in the last years and the mortality rate still remains close to 50%. Current chemotherapeutic treatments expose NB patients to high acute life-threatening toxicities, and cancer survivors have a significantly high risk to develop severe disabling chronic illnesses. In this context, the development of novel therapeutic approaches that combine high efficacy with low toxicity are urgently needed [1,2,3,4,5,6,7].

In previous works, we identified a set of non-coding (nc) RNAs, whose synthesis is driven by RNA polymerase (pol) III-type 3 promoters, involved in the modulation of the transcription of several genes [8,9,10,11]. One of these (named neuroblastoma differentiation marker 29, NDM29) maps in 11p15.3, an oncosuppressive region, whose deletion has been previously associated to NB development [12,13]. In agreement with this observation, over-expression of NDM29 reduces the malignancy of SKNBE2 NB cells. The number of tumor developed in SKNBE2 cell xenografted mice is inversely correlated with the level of NDM29 expression; moreover, within the tumor masses a significant reduction in the content of tumor-initiating/stem-like cells and the induction of neural differentiation of the tumor cells were observed [12,14,15]. From a drug response point of view, NDM29 expression potentiates the antiproliferative activity of metformin [16], an antidiabetic drug endowed with antitumor activity [17]. Thus, the overexpression of NDM29 may represent a valuable novel therapeutic approach.

In the attempt to identify pharmacological inducers of NDM29 expression using a drug repositioning approach [18], we carried out a screening of a drug library for membrane-permeable molecules (Prestwick Chemical Library, Illkirch, France) and identified two compounds able to powerfully increase the expression of NDM29 in NB cells. Interestingly, these compounds (perhexiline maleate and fendiline hydrochloride) are both antianginal drugs [19]. We previously reported that the pharmacological up-regulation of NDM29 by perhexiline maleate treatment drives the differentiation of NB cells, down-regulation of MDRs/ABC transporters, drug efflux proteins responsible for anticancer drug resistance, and confers an increased susceptibility to cisplatin cytotoxicity to malignant cells [19]. In fact, we showed that the co-administration of perhexiline maleate and cisplatin leads to a remarkable increase in cisplatin potency in a mouse model of NB, strongly enhancing animal survival rate, thus representing a possible new approach to improve cisplatin therapeutic efficacy [19].

In this work, due to the severe toxicological and pharmacokinetic limitations to the use of perhexiline in clinics, we investigated the effects of fendiline in combined treatment as enhancer of cisplatin antitumor effects. In this context, fendiline leads to a reduction of MDR expression driving to an increased susceptibility to cisplatin increasing its antitumor potency. This treatment results in a prolonged survival rate and a reduction of tumor mass growth in NB both in vitro and in vivo models.

## 2. Results

### 2.1. Fendiline Hydrochloride Selectively Induces NDM29 Expression in NB Cells

**Figure 1:**
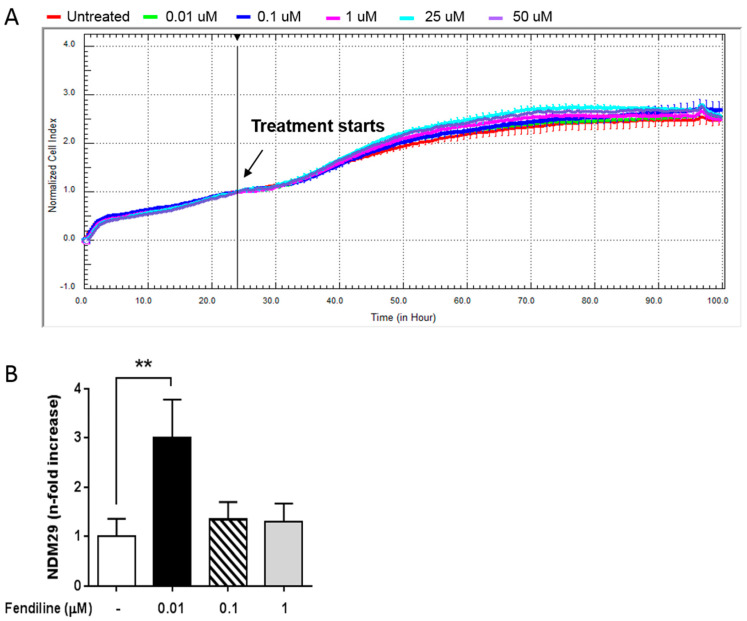
Fendiline hydrochloride treatment induces NDM29 expression and downregulates ABC transporters in neuroblastoma (NB) cells. (**A**) Effect of fendiline (0.01, 0.1, 1, 25, and 50 μM) on wild type SHSY5Y cell viability. Dose-response curves were obtained from cell index measured by the xCELLigence system. Cell index was recorded every 30 min and results for each concentration were the average of three replicates. Red line: untreated cells (DMSO); green line: 0.01 μM Fendiline; blue line: 0.1 μM fendiline; pink line: 1 μM fendiline; cyan line: 25 μM fendiline; purple line: 50 μM fendiline. (**B**) Expression levels of NDM29 in SHSY5Y cells either treated or untreated with fendiline (0.01, 0.1, and 1 μM), and measured by real-time RT-PCR. Values are reported as the mean ± SD. ** indicates *p* ≤ 0.01; two-tailed Student’s *t* test was applied.

In the present work, we investigated the effects of NDM29 overexpression induced by fendiline as a possible novel approach for NB therapy [19]. First, we dynamically monitored the possible toxic effects of fendiline for SH-SY5Y NB cells and its pharmacological window of activity using the xCELLigence system. To achieve this, we treated SH-SY5Y cells with increasing concentrations of the drug (0.01–50 μM) and compared normalized cell index curves to the untreated control cells. We found that fendiline exert neither antiproliferative nor toxic effect in SH-SY5Y cells, even at the highest concentration used (Figure 1A). Moreover, cell morphology was not affected by fendiline concentrations up to 1 μM in prolonged time-course experiments (24, 48, 72 h) (Supplementary Figure S1A).

Next, we assessed by qRT-PCR the effect of fendiline (0.01–1 μM) on NDM29 ncRNA expression. We observed that in vitro treatment with 0.01 μM fendiline for 24 h caused the highest increase of NDM29 levels (Figure 1B). Therefore, this concentration was chosen for all the subsequent experiments.

### 2.2. Fendiline Hydrochloride Reduces ABC Transporter Expression Increasing the Susceptibility of NB Cells to Cisplatin

**Figure 2:**
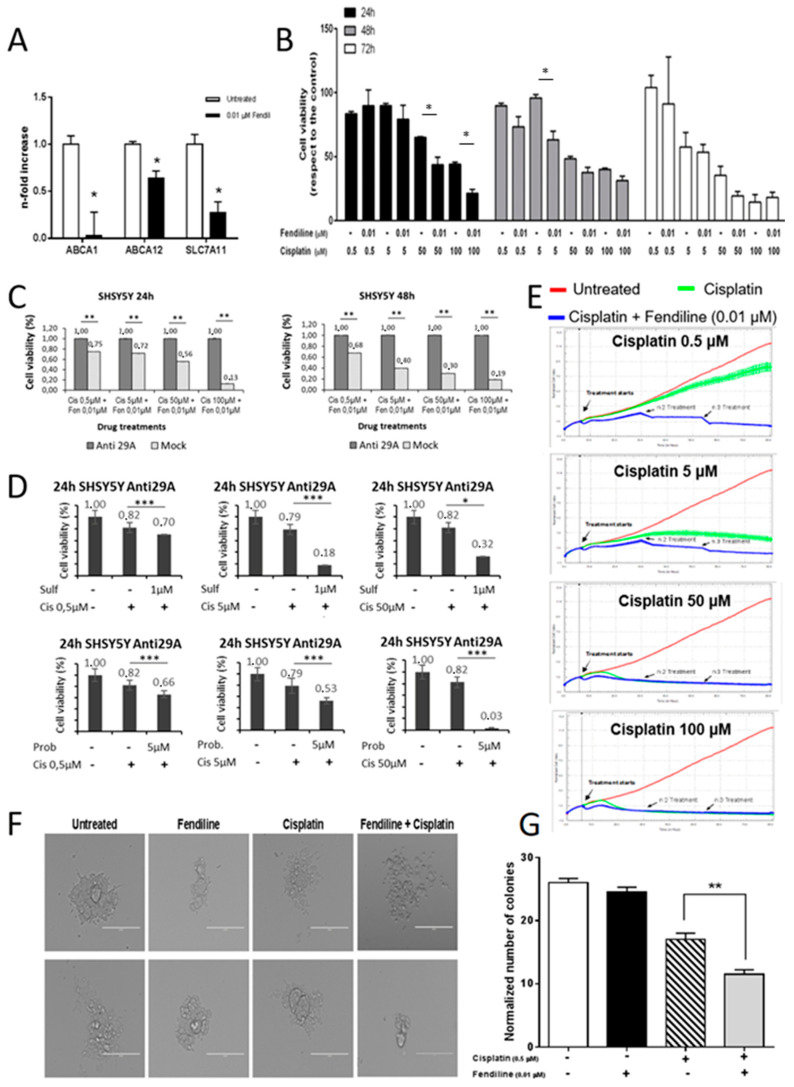
Susceptibility of NB cells to cisplatin is increased by fendiline hydrochloride. (**A**) Real-time RT-PCR quantification of ABC transporters’ mRNA level (ABCA1, ABCA12, and SLC7A11) in wild type SHSY5Y cells, either treated or untreated with 0.01 μM fendiline for 24 h. Values are reported as the mean ± SD. * indicates *p* ≤ 0.05, ** indicates *p* ≤ 0.01; two-tailed Student’s *t* test was applied. (**B**) Effects of 0.01 μM fendiline and cisplatin (0.5, 5, 50, 100 μM) or their combination on SHSY5Y cell viability after 24, 48, and 72 h of treatment, as detected by MTT assay. Values are expressed as the mean ± SD. (*) indicates *p* < 0.05; two-tailed Student’s *t* test was performed between the group treated with cisplatin at different concentrations and the group with cisplatin in combination with fendiline. (**C**) Cell viability measured by MTT assay after 24 and 48 h of treatment with cisplatin and Fendiline. Results were reported as the values of each sample previously normalized to its untreated control. (**D**) Demonstration of the dependence on the efflux pumps of the effects observed in the treatments. Sulf: sulfalazine. Prob: probucol. * indicates *p* ≤ 0.05, *** indicates *p* ≤ 0.001 (**E**) Kinetics of cytotoxicity responses for cisplatin (0.5, 5, 50, 100 μM) in wild type SHSY5Y cells, daily treated or untreated with 0.01 μM fendiline, monitored by RT-CES system. Cell index was recorded every 30 minutes and results for each concentration were expressed as the average of three replicates. Data were normalized to the time the compound was added. Red line: untreated cells (DMSO); green line: cisplatin; blue line: fendiline/cisplatin combination. (**F**) Colony forming capability of wild type SHSY5Y cells to form colonies in methylcellulose in the presence of different treatments (0.01 μM fendiline and/or 0.5 μM cisplatin). Two representative pictures of colonies at day 12 after seeding are reported for each experimental condition (0.01 μM fendiline and/or 0.5 μM cisplatin). Magnification 20×. Scale bar: 200 μm. (**G**) Statistical analysis of two independent experiments are reported as the mean ± SD (*n* = 5 microscope fields for each treatment). ** indicates *p* ≤ 0.01; two-tailed Student’s *t* test was applied.

We previously demonstrated that ABC transporters are down-regulated in NB cells overexpressing NDM29 ncRNA [19]. Hence, to verify whether also fendiline treatment may cause this effect, we measured by qRT-PCR the expression level of three ABC transporters involved in cell refractoriness to chemotherapeutics commonly used for the treatment of NB (5FU, cisplatin, and doxorubicin): ABCA1 (ATP-binding cassette A1), ABCA12 (ATP-binding cassette A12), and SLC7A11 (Solute Carrier Family 7 member 11). The treatment of SH-SY5Y cells with fendiline (0.01 μM) leads to a significant decrease of the mRNA for these efflux pumps (*p* ≤ 0.05), displaying a maximal activity on ABCA1, whose expression was almost abolished (Figure 2A). Therefore, we hypothesized that by reducing the activity of drug efflux pumps, fendiline treatment may induce a more efficient cisplatin intracellular accumulation causing an increased potency of its anticancer effects. In order to test this hypothesis, we measured SH-SY5Y cell viability by MTT assay, after treatment with increasing cisplatin concentrations (0.5–100 μM), in the presence or absence of fendiline (0.01 μM) (Figure 1B). In the absence of fendiline, cisplatin inhibits NB cell proliferation in a dose- and time-dependent manner, being statistically significant at the concentration of 100 µM after 24 h of treatment, at 50 µM after 48 h, and reaching a maximum of efficacy after 72 h (Supplementary Figure S1C).

The combination treatment with cisplatin and fendiline, which per se does not affect SH-SY5Y cell viability (Figure 1A, Supplementary Figure S1A,B), increased NB cell susceptibility to cisplatin. In the presence of fendiline, cisplatin cytotoxicity was statistically significant (*p* ≤ 0.05) at the concentration of 50 µM after only 24 h of treatment, and only 5 µM of cisplatin were required to cause a statistically significant cytotoxicity after 48 h (Figure 2B). In detail, the co-administration of fendiline and cisplatin caused a reduction of cell viability by 32% and 51% after 24 h of treatment with 50 and 100 μM cisplatin, respectively, and a 34% reduction of cell viability, compared to cisplatin alone with 5 μM cisplatin after 48 h of treatment (Figure 2B). As expected, a minor increase in cisplatin efficacy by fendiline co-treatment was observed after 72 h of treatment, since in these experimental conditions a maximal cytotoxicity by cisplatin alone was already detected (Figure 2B).

To assess whether the effects of fendiline and cisplatin co-administration are exerted via NDM29 ncRNA, we stably transfected SHSY5Y cells with a plasmid which permanently expresses a siRNA for NDM29. The selected clone (Anti-29A) was characterized in previously reported studies and shows a significantly lower levels of NDM29 ncRNA than both Mock-transfected and wt SHSY5Y cells [12].

As shown in Figure 2B, the combined treatment with cisplatin and fendiline for 24 and 48 h more effectively reduced cell viability in Mock cells (displaying a fendiline-dependent NDM29 overexpression as in wt cells, data not shown) than in Anti-29A cells (expressing very low levels of NDM29) demonstrating that induction of NDM29 ncRNA plays a pivotal role in the determination of the increased sensitivity of the cells to cisplatin (Figure 2C).

To demonstrate that fendiline sensitization to cisplatin cytotoxicity was dependent on the NDM29 down-regulation of efflux pumps, we used two validated inhibitors of the activity of ABCA1 and SLC7A6 pumps, probucol and sulfasalazine, respectively [20,21], and measured cisplatin effects in the absence of NDM29 upregulation. These experiments showed that the pharmacological inhibition of these pumps mimics the effects induced by fendiline treatment, clearly suggesting that the downregulation of their expression induced by NDM29 is responsible for the increased cisplatin activity (Figure 2D).

To strengthen these results, we investigated the effects of the combined treatment on NB cell proliferation also using the xCELLigence system. In these experiments, as fendiline has a short half-life, it was daily added at the concentration of 0.01 μM to the cell medium. Again, although the treatment with fendiline alone was ineffective (see Supplementary Figure S1D), and the exposition to cisplatin alone exerted the expected dose-dependent inhibition of cell proliferation (Supplementary Figure S1D), the combined treatment significantly increased cisplatin impairment of proliferation rate of tumor cells, mainly when used at low concentrations. Indeed, the modest reduction of cell viability induced by 0.5 μM cisplatin alone was strongly potentiated by the co-administration with fendiline (Figure 2E).

Moreover, we also observed that a higher pharmacological effect was induced by the cisplatin/fendiline combined treatment even at the highest cisplatin concentrations (50 μM and 100 μM) at which it is per se already highly toxic. In the absence of fendiline, cisplatin needs about 16 h to induce NB cell death, while, when co-administered with fendiline, cisplatin antiproliferative activity occurred with a very short lag-time (Figure 2E and Supplementary Figure S1E). These results suggest that the co-treatment with fendiline and cisplatin increases NB cell sensitivity to cisplatin-induced cytotoxicity enhancing the potency of the anticancer drug.

Since we previously reported that the overexpression of NDM29 reduces the tumorigenic potential of NB cells [12], we investigated the effect of the combined treatment with fendiline and cisplatin on the clonogenic activity of SH-SY5Y cells, by methylcellulose clone formation assay. We observed no changes in both colony morphology, size, and average number of colonies per microscope field (as normalized to controls) in cells treated with 0.01 μM fendiline, confirming that fendiline does not exert per se anticancer effects (Figure 2F,G). In the same experimental conditions, the clonogenic potential of NB cells, impaired by 0.5 μM cisplatin as compared to control cells (−34%, *p* ≤ 0.01), was further reduced by the administration of the combination fendiline/cisplatin (−55%, *p* ≤ 0.001, as compared to the control and −32%, *p* ≤ 0.01, vs. cisplatin alone) (Figure 2G). Altogether, these results suggest that fendiline potentiates the cisplatin inhibition of clonogenic potential of NB cells.

### 2.3. Cisplatin and Fendiline Hydrochloride Act Synergistically in a Combo-Treatment

In order to determine the mechanism of this drug interaction in the potentiating effects of fendiline on cisplatin antitumor activity (synergism or additivity), we performed the multiple drug effect analysis developed by Chou and Talalay [22], using the combination index method [22,23,24,25]. For this purpose, we measured cell viability impairment, using the MTT assay to calculate the CI values for fendiline and cisplatin concentrations corresponding to the respective EC_50_ values (see Figure 2).

We found that after 24 h of treatment, the CI value was 0.529 indicating a strong synergic interaction between the two drugs in combined therapy at all the doses tested. After 48 h, the CI value was 0.769 in which the increased toxicity observed after prolonged treatment induced by cisplatin alone reduced the possibility of fendiline to potentiate the response. Therefore, these results indicate that fendiline increases the susceptibility of cells to cisplatin synergizing with its cytotoxic effects.

### 2.4. Co-Administration of Fendiline Hydrochloride Markedly Strengthens Cisplatin Antitumor Activity In Vivo

Based on these in vitro results, we performed in vivo experiments to investigate whether fendiline co-treatment might enhance the chemotherapeutic efficacy of cisplatin in vivo. To this aim, after subcutaneous xenografting of NOD-SCID mice with SKNBE2 NB cells, we treated the animals with fendiline and cisplatin, alone or in combination, and assessed both tumor growth rate and overall animal survival.

**Figure 3:**
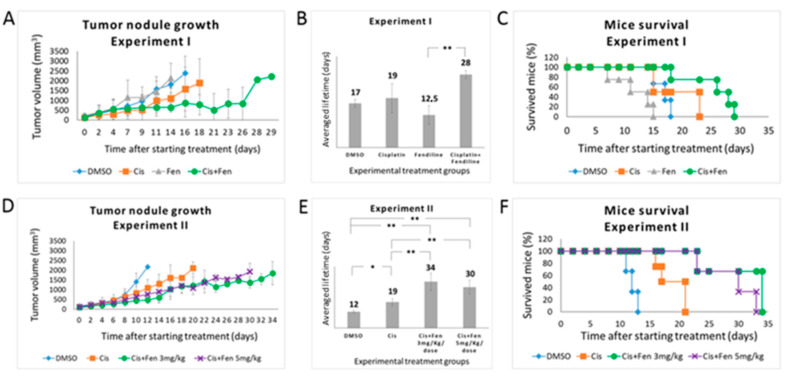
Fendiline hydrochloride in co-administration with cisplatin reduces the growth of tumor nodules in vivo and increases mice survival. (**A**) Tumor nodules growth rate in NOD-SCID mice treated with different drug combinations. In the first experiment, mice were randomized to receive the following treatments: vehicle (1% DMSO) once a day for 5 days/week; fendiline (3 mg/kg/dose), once a day for 5 days/week; cisplatin (5 mg/kg/dose), weekly; fendiline (3 mg/kg/dose), once a day for 5 days/week in co-administration with cisplatin, weekly. Mice were sacrificed when the tumor size reached 2.2 cm^2^. (**B**,**C**) Statistical analysis of Kaplan–Meier plots of the first in vivo experiment. Log Rank test showed a statistically significant difference (*p* < 0.05) between the following groups: (i) DMSO vs. fendiline; (ii) fendiline co-administered with cisplatin vs. DMSO; (iii) fendiline co-administered with cisplatin vs. cisplatin; iv) fendiline co-administered with cisplatin vs. fendiline. (**D**) Tumor nodules growth rate in NOD-SCID mice treated with different drug combinations. In the second experiment, mice were randomized to receive the following treatments: vehicle (1% DMSO), once a day for 7 days/week; cisplatin (5 mg/kg/dose), weekly; fendiline using either 3 mg/kg/dose or 5 mg/kg/dose, once a day for 7 days/week in co-administration with cisplatin, weekly. (**E**,**F**) Log Rank test of Kaplan–Meier plots showed a statistically significant difference (*p* < 0.05) between the following groups: (i) DMSO vs. cisplatin; (ii) fendiline 3 mg/kg/dose co-administrated with cisplatin vs. DMSO; (iii) fendiline 5 mg/kg/dose co-administrated with cisplatin vs. DMSO; (iv) fendiline 3 mg/kg/dose co-administrated with cisplatin vs. cisplatin; (v) fendiline 5 mg/kg/dose co-administrated with cisplatin vs. cisplatin. Values are reported as the mean ± SD. * indicates *p* ≤ 0.05, ** indicate *p* ≤ 0.01; two-tailed Student’s *t* test was applied.

In the first experiment, we administered a combination of fendiline (3 mg/kg/dose, daily for 5 days a week) and cisplatin (5 mg/kg/dose, once a week). Three parallel groups of mice were used as experimental controls and treated with cisplatin alone (5 mg/kg/dose), fendiline alone (3 mg/kg/dose), and DMSO (1% in saline solution as vehicle). Fendiline- and DMSO-treated control groups rapidly developed tumors, reaching the established tumor mass threshold of 2.2 cm^3^ after 14/16 days, confirming that fendiline does not exert direct antitumor effects in NB as a single agent (Figure 3A). These two groups did not show statistically significant differences in both tumor mass growth and progression-free survival: PFS was 12.5 and 17 days for fendiline- and DMSO-treated groups, respectively (*p* = 0.051) (Figure 3B). In the same conditions, cisplatin-treated group reached the tumor mass threshold after 19 days, thus showing only a moderate antitumor effect, again not significantly different from both fendiline and DMSO (*p* = 0.059 and *p* = 0.944 as compared to fendiline and DMSO groups, respectively) (Figure 3B).

Conversely, the combined treatment significantly improved PFS of 28 days (evidencing that the co-administration of fendiline enhances cisplatin efficacy, although a statistical significance was obtained, in this experimental setting, only in comparison with fendiline alone *p* = 0.093, *p* = 0.0011, and *p* = 0.068 as compared to cisplatin, fendiline, and DMSO groups, respectively) (Figure 3A,B). On the contrary, the Log Rank test performed on Kaplan–Meier data yielded *p* < 0.05 for the combined therapy as compared to all other groups (Figure 3C).

To better analyze the in vivo effects of the combined treatment with fendiline and cisplatin, we performed a second set of experiments. The design of this experiment differed from the former for the timing of fendiline delivery (which was daily administered for 7 days a week, to increase the antitumor effects) and for the use of two concentrations of this drug (3 and 5 mg/kg/dose) in combination with cisplatin. In terms of tumor growth rate and PFS, results from control groups overlapped those from the first experiment (Figure 3D,E). In this condition, cisplatin/fendiline-treated group showed a decreased tumor growth rate and a significantly improved PFS, with no remarkable differences between the two fendiline dosages (PFS were 34 and 30 days at 3 mg/kg/dose and 5 mg/kg/dose of fendiline, respectively), reaching in both cases statistically significant differences with respect to cisplatin alone and control (DMSO-treated) groups (*p* < 0.01). As expected, the effect of cisplatin alone was significant (*p* < 0.05) versus DMSO controls. On the contrary, the two fendiline concentrations within the combo therapy were not significantly different from each other (*p* = 0.306) (Figure 3D,E). The Log Rank test performed on Kaplan–Meier data from both in vivo experiments yielded significant differences (*p* < 0.05) for both dosage of combined therapy as compared to both control and cisplatin alone groups (Figure 3F). Again, these results indicated that the administration of fendiline increases the anticancer efficacy of cisplatin, although it requires a continuous administration, likely due to its short acting in vivo activity. The expression level of ABCA1 and SLC7A11 has been evaluated in three independent tumor nodules treated with DMSO or fendiline/cisplatin; results showed a significant inhibition of their transcription (see Supplementary Figure S3).

### 2.5. Co-Administration of Fendiline Hydrochloride and Cisplatin Induces Apoptosis and Reduces the Fraction of GD2+ NB Cells

To study the effects of the combinational therapy on NB tumor masses and the possible correlation between reduced tumor growth and specific modifications of the NB cell phenotype, we carried out histological analyses of the tumors explanted from both treated and control mice. Combination treatment caused a significant reduction of tumor nodule volume and weight as compared to all the other experimental groups (Supplementary Figure S2).

At a macroscopic analysis, tumor nodule morphology derived from cisplatin/fendiline-treated mice showed strong peculiar alterations of consistency and color with respect to the cisplatin-treated and the control groups, showing a possible poor vascularization associated to large macroscopic necrotic areas.

**Figure 4:**
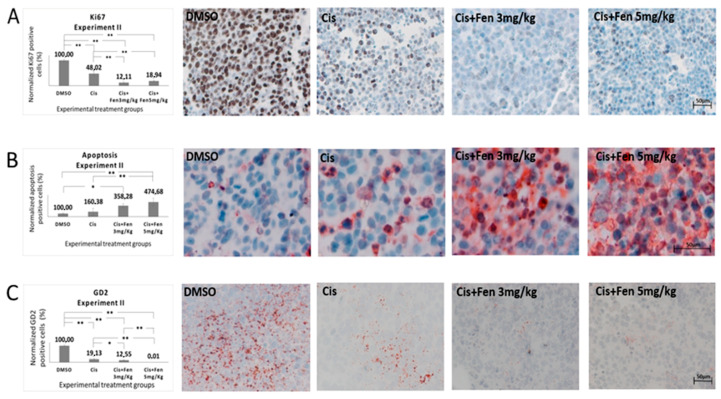
Fendiline hydrochloride in co-administration with cisplatin induces apoptosis and reduces GD2+ NB cells. Representative bright-field microscopy imaging of stained sections from subcutaneous tumors in treated and untreated mice. Panel (**A**) shows how the combined therapy reduces the expression of the cell proliferation marker Ki67 in tumors from treated mice. Panel (**B**) shows how the combined therapy increases apoptotic cell rate in tumor tissues. Panel (**C**) show GD2 immunopositive tissues. GD2 was used as NB tumor aggressiveness marker showing a positive correlation with the concentration of fendiline used. Values are reported as the mean ± SD. * indicates *p* ≤ 0.05, ** indicates *p* ≤ 0.01; one-way ANOVA with post-hoc Tukey HSD were applied. Scale bar: 50 μm.

In order to better define these alterations, we stained tumor nodule sections from experiments II with Ki67 Ab, to identify differences in the proliferation rate of NB cells within the nodules. Results showed a significant decrease of Ki67^+^ cells (*p* < 0.01) in tumor tissues from mice treated with the combined therapy, as compared to those treated with cisplatin alone. Statistically significant differences were also observed between the combinational treatment with both concentrations of fendiline and controls (Figure 4A).

Next, we analyzed, in the same sections, the presence of apoptotic cells, by TUNEL assay. We found that both fendiline concentrations co-administered with cisplatin led to a significant increase in the percentage of apoptotic cells (*p* < 0.01) with respect to control group; the combination treatment with 5 mg/kg fendiline and cisplatin showed a significantly higher content of apoptotic cells (*p* < 0.01) even when compared to cisplatin alone, thus evidencing that fendiline increases the cytotoxic effect of cisplatin in anticancer therapy (Figure 4B).

Finally, to assess the effects of the fendiline/cisplatin treatment on NB aggressiveness, we measured the fraction of GD2^+^ cells, in the tumor sections from in vivo experiment II. We observed a statistically significant decrease of GD2^+^ cells in NB nodules treated with the combo-therapy with both fendiline doses (3 mg/kg: *p* < 0.05; 5 mg/kg: *p* < 0.01), as compared to those treated with cisplatin alone. These results were confirmed in the third experiment in vivo, in which the combo-therapy significantly decreased GD2^+^ cells (*p* < 0.01) with respect to all the other experimental groups. Altogether, these results indicate that the co-administration of fendiline reduces the rate of GD2^+^ cells in NB nodules, possibly lowering the tumorigenic potential of the nodules (Figure 4C).

## 3. Discussion

The high heterogeneity of NB nodules, mainly due to the presence of cells at different stages of differentiation and transformation, renders this tumor difficult to eradicate by current cytotoxic therapies, and relapses are often observed. Indeed, in NB tumor masses, several cells are resistant to chemotherapeutics also when used at high and toxic dosages, which are extremely dangerous for patients. Recently, we reported the identification of two small molecules that were potentially able to induce the expression of NDM29 ncRNA, which, downregulating ABC transporters expression in cancer cells, render NB more susceptible to co-administered chemotherapeuticals [12,14,15]. This effect was initially demonstrated in preclinical studies in vivo using one of the two drugs, perhexiline maleate co-administered with cisplatin in NB mouse xenografts [19]. However, concerns about safety of this compound limit its clinical development as novel adjuvant antitumor therapy.

In the present work, we demonstrate that also fendiline potentiates antitumor effects of cisplatin when co-administered with cisplatin in mice xenografted with NB cells. Fendiline anticancer activity was also reported by other groups, which reported that this drug interferes with the activation of ADAM 10 [26], blocks K-RAS signaling transmission [27], or evokes Ca^++^-triggered cell death in human oral, hepatoma, and female bladder transitional carcinoma cells [28,29,30]. However, in these previous works, fendiline was studied as a possible anticancer drug being administered alone. In addition, further studies supported the possible use of fendiline to enhance the efficacy of specific anticancer molecules in combined therapies of pancreatic ductal adenocarcinoma cells [31].

Here, we report that fendiline is per se ineffective in inducing antitumor effects in NB cells in vitro and in vivo, but, through the inhibition of the expression of several transporters linked to multidrug resistance (ABCA1, ABCA12, SLC7A11), it greatly potentiates the antitumor efficacy of cisplatin. It is worth to note that fendiline effects are not concentration-dependent and showed a reduced activity at the highest concentrations tested. Currently, we do not have an explanation for this evidence at molecular level, but in all the in vitro experiments we used a fendiline concentration which constantly increased NDM29 expression. However, in a clinical translation perspective, although a direct correlation between in vitro concentrations and in vivo doses cannot be extrapolated, these data should be considered if clinical trials in humans will be developed. On the other hand, we observed that a better antitumor response was obtained by daily administration of fendiline (compare in vivo experiment I and II), also allowing a high efficacy at low doses. The absence of significant toxicity of the drug that is extensively metabolized in similar conditions was previously demonstrated in humans [32]. Apparently, treatment with fendiline alone caused a slight reduction of mice survival as compared to control group. However, we have to remark that the difference was not statistically significant and likely reflect the variability of the in vivo experiments, especially when small groups of animals are used. The low toxicity of fendiline when used as single treatment, is indeed clearly demonstrated in all the in vitro experiments.

The inhibition of NB growth induced by the co-administration of fendiline and cisplatin was characterized by a strong synergism, as observed both in vitro and in vivo. In both experimental conditions, the effect was higher than that of cisplatin alone and, remarkably, observed in the absence of a direct antitumor activity of fendiline. In particular, in vivo experiments showed that the administration of fendiline powerfully increases the cisplatin pro-apoptotic activity. Importantly, the combined therapy targets with high-specificity GD2-positive NB cells, which are the tumor initiating cell (TIC) component within the tumor mass and the main drug resistant subpopulation responsible of tumor relapse. Therefore, in the light of these results, the combined fendiline/cisplatin treatment is not only advantageous for the potentiation of cisplatin effects but also for its specificity for the TIC fraction. Thus, it is reasonable to hypothesize that during NB patients’ treatment with cisplatin, the co-administration of fendiline, decreasing TIC content in the nodules, might reduce significantly the rate of tumor relapse and prolong the progression-free survival of patients. In conclusion, we propose that the combined treatment with fendiline and cisplatin could represent a possible novel therapeutic protocol aimed at increasing the survival rate of NB patients and reducing the risk of relapses to be tested in clinical setting.

## 4. Materials and Methods

### 4.1. Screening Assay

We screened on SH-SY5Y cells stably expressing luciferase obtained from the Prestwick Chemical Library (Illkirch, France). In total, 45 × 10^3^ cells were transfected and processed as described elsewhere [19].

### 4.2. Cell Cultures

SKNBE2 and SHSY5Y neuroblastoma cells were provided by the cell bank of the National Institute of Cancer Research (IST) Genoa, Italy and obtained from ECACC.

SKNBE2 were maintained in RPMI 1640 medium (EuroClone, Devon, UK), 10% FBS (GIBCO, S. Giuliano Milanese, Milan, Italy), 2 mM L-glutamine (EuroClone), 100 U/mL penicillin–streptomycin (EuroClone). SHSY5Y cells were cultured in Dulbecco’s modified Eagles medium (DMEM) (EuroClone), 10% FBS (GIBCO), 2 mM L-glutamine (EuroClone), and 100 U/mL penicillin-streptomycin (EuroClone).

### 4.3. qPCR Analysis

Total RNA was extracted from samples using TRIzol reagent (Invitrogen, Carlsbad, CA, USA) according to the manufacturer’s protocol and subjected to reverse transcription by Transcriptor First Strand cDNA Synthesis Kit (Roche, Mannheim, Germany), containing random hexamers. Total RNA was measured by qPCR using PE ABI PRISM@ 7700 Sequence Detection System (Applied Biosystem, Thermo Fisher Scientific, Waltham, MA, USA) and SYBR Green method. Forward and reverse primer sequences were: NDM29 Forward (GGCAGGCGGGTTCGTT) and Reverse (CCACGCCTGGCTAAGTTTTG); ABCA1 Forward (GCGAGTACTTCGTTCCAACATG) and Reverse (TCGGGAAGGGAGATGTAGAGTTT); ABCA12 Forward (ATGCATCTGCCCAGAAGTGTT) and Reverse (GGTGTGTTCATTCGGTTGCTT); SLC7A11 Forward (TCCATGAACGGTGGTGTGTTT) and Reverse (ACCCTCTCGAGACGCAACAT); GAPDH Forward (GAAGGTGAAGGTCGGAGTC) and Reverse (GAAGATGGTGATGGGATTTC). No-template controls, containing no cDNA, were run under the same conditions for each gene. Transcript levels were determined from the relative standard curve constructed from stock cDNA dilutions, after normalization to housekeeping gene levels. Targets relative quantification was then expressed as the n-fold quantity of the calibrator.

### 4.4. NDM29 Down-Regulation

In order to down-regulate NDM29 ncRNA, we designed an engineered microRNA (miRNA-NDM29; 5′TGCTGTTCAACAAGCAATAGCGTCTAGTTTTGGCCACTGACTGACTAGACGCTTGCTTGTTGAA-3′) against the NDM29 sequence following the BLOCK-iT Pol II miR RNAi Expression Vector kit guidelines (Invitrogen). We then cloned tmiRNA-NDM29 in the pCEG vector. Control experiments were performed using either pCEG alone or the pCMMP retroviral GFP vectors.

### 4.5. xCELLigence System Cytotoxicity Assays

Cell proliferation and cytotoxicity was assessed monitoring cellular events in real time by measuring electrical impedance across interdigitated gold micro-electrodes integrated at the bottom of tissue culture plates by xCELLigence RTCA MP System (Roche, Mannheim, Germany). Cell-sensor impedance is expressed as an arbitrary unit called Cell Index. Briefly, SHSY5Y cells were seeded (7 × 10^4^ cells per well) in 100 μL of standard medium in E-plates (Roche), and compounds added after 24 h. Fendiline was dissolved in DMSO. DMSO controls were also performed. Cell proliferation was monitored for at least 72 h. Proliferation, spreading, and cell attachment were measured every 30 min producing time-dependent cell response dynamic curves. Different concentrations of fendiline (from 0.01 to 50 μM) and cisplatin (from 0.5 to 100 μM) were tested separately or in combination. Data analysis was performed by RTCA Software 1.2.

### 4.6. MTT Assay

Twenty-four hours after plating, cells were exposed to drugs to be tested: cisplatin (Accord, Italy), and/or fendiline hydrochloride (Sigma Aldrich, cat. Number F7265) and/or 1 µM sulfasalazine (Sigma Aldrich, cat. Number S0883) and/or 5µM probucol (Sigma Aldrich, cat. Number P9672), at 37 °C. At the end of the treatment, the medium was removed and cells were incubated for 1 h with 3-(4,5-Dimethylthiazol-2-yl)-2, 5-Diphenyltetrazolium Bromide (MTT) solution (2 mg/mL in PBS) (Sigma-Aldrich). In order to dissolve formazan crystals, after removing MTT 500 μL of 100% ethanol were added to each well, and absorbance was determined at 570 nm, using a reference filter at 670 nm. Cytotoxicity was expressed as percentage of viable cells compared to untreated cells.

### 4.7. Determination of Combination Index Values

MTT assay (method described above) was performed on SHSY5Y cell line. Drug-induced cytotoxic synergy was analyzed using the median-effect method [22,23], and expressed as the combination index (CI). CI describes the nature of drug–drug interactions that can be additive (CI = 1), antagonistic (CI > 1), or synergistic (CI < 1) for various concentrations [24,33]. CI values were calculated using CompuSyn software (ComboSyn Inc., Paramus, NJ, USA), following the method by Chou and Talalay [22] that further refines the value as moderate synergism (CI = 0.7–0.9), synergism (CI = 0.3–0.7), strong synergism (CI = 0.1–0.3), and very strong synergism (CI < 0.1) [22].

### 4.8. Methylcellulose Colony Formation Assay

A medium consisting of DMEM with 0.4% methylcellulose (Methocult H4100, StemCell Technologies, Vancouver, BC, Canada) supplemented with 10% FBS, 100 U/mL penicillin/streptomycin, and 2 mM L-glutamine was used to perform clonogenic assay. 500 SH-SY5Y cells were seeded in humidified 6-well plates and colonies were counted 12 days after plating. Images were acquired at 20× or 4× magnification on an EVOS FL digital inverted microscope (Advanced Microscopy Group, WA, USA). Data were recorded from two independent assays, both performed in duplicate. Each treatment data represent the average of five microscope fields [19].

### 4.9. Mice

Homozygous NOD-SCID mice were purchased from the Animal Facility of IRCCS Ospedale Policlinico San Martino (Genoa, Italy). Mice were tested at age ranging between 5 and 8 weeks. All experimental procedures involving animals were carried out in accordance with the guidelines of the European Community for the use and care of live animals, approved by the Italian Ministry of Health (D.lgs.vo 116/92) and by the Ethics Committee of the Animal Facility of IRCCS AOU San Martino-IST (protocol DGSAF 0001448-A). Efforts were made to minimize animal stress/discomfort.

### 4.10. In Vivo Tumorigenic Assays

SKNBE2 cells (5 × 10^6^) were resuspended in PBS and subcutaneously injected into the right side of NOD-SCID mice. Mice were then divided into several groups and two experimental therapeutic protocols were designed. Each experimental protocol consisted of 20 mice that were randomly divided into 4 treatment groups, 5 mice for each group. In the first experiment, three treatment groups were used as controls. Hence, treatment control groups were administered with either the vehicle (1% DMSO) or 5 mg/kg/dose cisplatin or 3 mg/kg/dose fendiline. The fourth group was treated with a combination of 3 mg/kg/dose fendiline and 5 mg/kg/dose cisplatin. The second experiment presented two controls group: DMSO and cisplatin 5 mg/kg/dose. The third and fourth groups were treated with a combination of 3 mg/kg/dose or 5 mg/kg/dose fendiline, respectively, and 5 mg/kg/dose cisplatin.

Cisplatin was administered by intraperitoneal injection once a week. Fendiline and DMSO were administered by gastric gavage once a day 5 days a week in the first protocol, and once a day for 7 days a week in the second one. All treatments began when the tumor mass reached a diameter of 5 mm. Tumor size was measured three times a week with calipers and tumor volume calculated by the following formula: length^2^ × width × π/6. Mice were sacrificed when tumor size was 2.2 cm^3^.

### 4.11. Immunohistochemical Staining

Five-micrometer thick sections were obtained from tumors explanted from mice from the previously described experiments. Immunochemistry detection of Ki67 and MCM2 was performed on sections fixed with 4% paraformaldehyde and paraffin-embedded. Antigen retrieval was obtained using 6.0 pH citrate buffer in a microwave oven, when required. The sections were immunostained using Ki67 antibody (1:200, SP6 NB600-1252, Novus Biologicals, Littleton, CO, USA) and MCM2 antibody (1:1000, MA5-15895, Thermo Scientific, Waltham, MA, USA) overnight at 4 °C. Immunochemistry detection of GD2 (1:300, MAB2052, Merk Millipore, Billerica, MA, USA) was conducted on frozen sections of tumors, embedded in Killik (05-9801, Bio-Optica, Milano, Italy).

The antibody complex was revealed with ImmPRESS HRP Reagent Kit (MP-75000, Vector, Burlingame, CA, USA) and Liquid DAB + Substrate Chromogen System (K3468, Dako, Carpinteria, 93013-USA) for Ki67 and MCM2, whereas GD2 was revealed using AEC+High Sensitivity Chromogen Ready-to-Use (K3461, Dako Carpinteria, 93013-USA). The sections were counterstained with modified Mayer hematoxylin and mounted in Glycergel Mounting Medium (C0563, Dako Carpinteria, 93013-USA).

In order to quantify DAB-immunohistochemical staining of MCM2, from each tumor sections 8 microscope fields were acquired (Axiovert 200M, Zeiss) at 20× magnification and the proportion of positive area determined by ImageJ software.

Images were converted in RGB and threshold was manually adjusted to localize DAB-stained areas of interest. The number of pixels within the hue range set was expressed as a percentage of the total selected area.

In order to quantify Ki67 immunohistochemical staining, 8 images per sample (representing central and peripheral tumor regions) were acquired using 20× objective. The percentage of DAB-stained nuclei with respect to the total nuclei (DAB and hematoxylin-stained) was calculated using ImmunoRatio program, that calculates the percentage of positively stained nuclear area (labeling index) by using a color deconvolution algorithm in order to separate the staining components. The ImmunoRatio is a free online application for automated image analysis to quantify Ki67 positive cells on immunostained slices [34,35].

### 4.12. Apoptosis Analysis

TUNEL assay, using the “In Situ Cell Death Detection Kit, Fluorescein” (Roche) was employed for the detection of apoptosis on tumor sections. For quantification of positive cells, 8 randomly chosen microscope fields were captured at 40 × magnification and counted.

### 4.13. Statistical Analysis

Results are expressed as mean ± Standard Deviation. Statistical significance of observed differences among different experimental groups was calculated using a two-tailed unpaired Student’s *t* test for in vitro methods and using One-way ANOVA with post-hoc Tukey HSD (Honestly Significant Difference) for in vivo tests and derived samples. A *p*-value ≥ 0.05 was considered to be statistically significant. For survival studies, Kaplan–Meier curves were plotted and compared using the log-rank test. The statistical calculations were performed with GraphPad Prism 6.0 (GraphPad Software, La Jolla, CA, USA).

## Supplementary Materials

The following are available online, Figure S1: Fendiline hydrochloride treatment induces NDM29 expression and downregulates ABC transporters in NB cells, Figure S2: Susceptibility of NB cells to cisplatin is increased by fendiline hydrochloride. Figure S3: Real Time RT-PCR analysis of ABCA1 and SLC7A11 transcription modulation in tumor nodules following cisplatin/fendiline administration.
