# Supplementary material for: Co-Administration of Fendiline Hydrochloride Enhances Chemotherapeutic Efficacy of Cisplatin in Neuroblastoma Treatment
Source: Molecules. 2020 Nov 10;25(22):5234. doi: 10.3390/molecules25225234 (PMC7698186; doi:10.3390/molecules25225234)
Supplement: Supplementary file 1 [file molecules-25-05234-s001.pdf]

Supplementary Material

Co-administration of Fendiline Hydrochloride Enhances Chemotherapeutic Efficacy of Cisplatin in Neuroblastoma Treatment

Antonella Brizzolara [1,†], Patrizia Garbati [2,†], Serena Vella [3,4,†], Matilde Calderoni [2], Alessandro Quattrone [5], Gian Paolo Tonini [6], Mario Capasso [7,8,9], Luca Longo [10], Raffaella Barbieri [2], Tullio Florio [1,11] and Aldo Pagano [1,2,*]

[1] IRCCS AOU San Martino Polyclinic Hospital, 16132 Genova, Italy; antonella.brizzolara@yahoo.it (A.B.); tullio.florio@unige.it (T.F.)

[2] Department of Experimental Medicine (DIMES), University of Genova, 16126 Genova, Italy; p.r.garbati@gmail.com (P.G.); calderoni.matilde@gmail.com (M.C.); raffaella.barbieri@edu.unige.it (R.B.)

[3] Department of Laboratory Medicine and Advanced Biotechnologies, Institute of Hospitalization and Care of a Scientific nature - Mediterranean Institute for Transplantation and Highly Specialized Therapies (IRCCS- ISMETT), 90127 Palermo, Italy; vellaserena@gmail.com

[4] Anemocyte S.r.l., 21040 Gerenzano, Italy

[5] Laboratory of Translational Genomics, Centre for Integrative Biology, University of Trento, 38123 Trento, Italy; alessandro.quattrone@unitn.it

[6] Neuroblastoma Laboratory, Pediatric Research Institute, The "Città della Speranza" Foundation, 35128 Padua, Italy; gp.tonini@irpcds.org

[7] Department of Molecular Medicine and Medical Biotechnology, University of Naples Federico II, 80145 Naples, Italy; mario.capasso@unina.it

[8] CEINGE Biotecnologie Avanzate, 80131 Naples, Italy

[9] SDN Research Institute Diagnostics and Nuclear, 80133 Naples, Italy

[10] Lung Cancer Unit, Division of Medical Oncology II, IRCCS San Martino Polyclinic Hospital, 16132 Genova, Italy; longo.luk@gmail.com

[11] Department of Internal Medicine (DIMI), University of Genova, 16126 Genova, Italy

* Correspondence: aldo.pagano@unige.it; Tel.: +39-010-5558213

†These authors contributed equally to this work.

Academic Editor: M. Amparo F. Faustino

Received: 8 September 2020; Accepted: 3 November 2020; Published: date

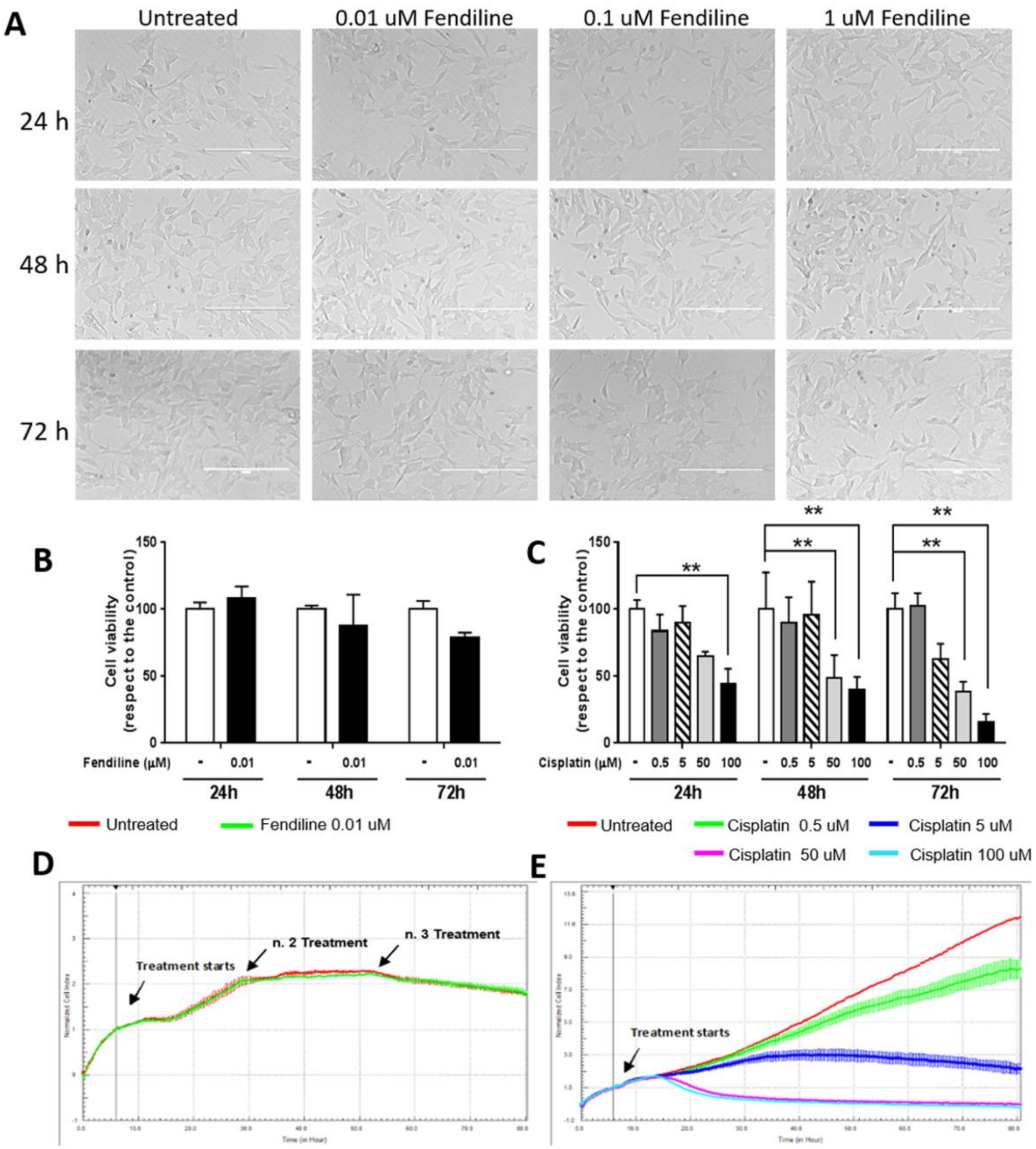

Figure S1. Fendiline hydrochloride treatment induces NDM29 expression and downregulates ABC transporters in NB cells. **A** Phase contrast microscopy of wild type SHSH5Y cells cultured in presence or absence of Fendiline hydrochloride (0.01, 0.1 and 1 μM). Cells were monitored at various times up to 72 hours by phase contrast microscopy at 20X magnification. Scale bar: 200μm. Effects of 0.01 μM fendiline **B** or 0.5-100 μM cisplatin **C** on SHSY5Y cell viability (MTT assay) after 24, 48 and 72 hours of treatment. Values are reported as the mean ± SD. (**) indicates p ≤ 0.01. Effects of 0.01 μM fendiline administered as a daily treatment **D** and of 0.5-5-50-100 μM cisplatin **E** on wild SHSY5Y cell viability. Dose-response curves were obtained from cell index measured by the xCELLigence system. Results for each concentration were the average of three replicates. Data were normalized to the time the compound was added. Various treatments are indicated by different colors.

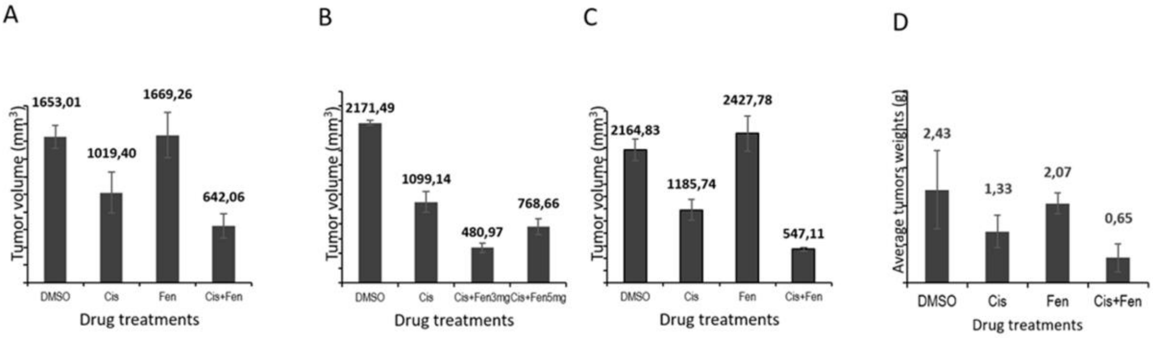

Figure S2. Susceptibility of NB cells to cisplatin is increased by fendiline hydrochloride. Averaged tumor nodules volume and weight after 12 days of treatment with different drug combinations (reported in Materials and Methods section) in three different experiments. **A** Experiment 1; **B** Experiment 2; **C** Experiment 3; **D** Averaged weight of tumor nodules. Results show a remarkable difference among the experimental groups in tissue density.

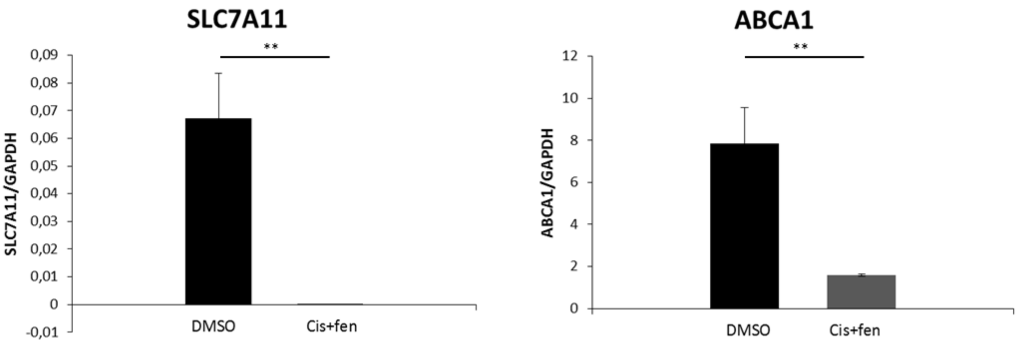

(**a**) (**b**)

Figure S3. Real Time RT-PCR analysis of SLC7A11 (**a**) and ABCA1 (**b**) transcription modulation in tumor nodules following cisplatin/fendiline administration.
